# Exhausted CD8 T Cells Downregulate the IL-18 Receptor and Become Unresponsive to Inflammatory Cytokines and Bacterial Co-infections

**DOI:** 10.1371/journal.ppat.1002273

**Published:** 2011-09-29

**Authors:** Jennifer T. Ingram, John S. Yi, Allan J. Zajac

**Affiliations:** 1 Department of Biology, University of Alabama at Birmingham, Birmingham, Alabama, United States of America; 2 Department of Microbiology, University of Alabama at Birmingham, Birmingham, Alabama, United States of America; University of Pennsylvania, United States of America

## Abstract

During many chronic infections virus-specific CD8 T cells succumb to exhaustion as they lose their ability to respond to antigenic activation. Combinations of IL-12, IL-18, and IL-21 have been shown to induce the antigen-independent production of interferon (IFN)-γ by effector and memory CD8 T cells. In this study we investigated whether exhausted CD8 T cells are sensitive to activation by these cytokines. We show that effector and memory, but not exhausted, CD8 T cells produce IFN-γ and upregulate CD25 following exposure to certain combinations of IL-12, IL-18, and IL-21. The unresponsiveness of exhausted CD8 T cells is associated with downregulation of the IL-18-receptor-α (IL-18Rα). Although IL-18Rα expression is connected with the ability of memory CD8 T cells to self-renew and efflux rhodamine 123, the IL-18Rα^lo^ exhausted cells remained capable of secreting this dye. To further evaluate the consequences of IL-18Rα downregulation, we tracked the fate of IL-18Rα-deficient CD8 T cells in chronically infected mixed bone marrow chimeras and discovered that IL-18Rα affects the initial but not later phases of the response. The antigen-independent responsiveness of exhausted CD8 T cells was also investigated following co-infection with *Listeria monocytogenes*, which induces the expression of IL-12 and IL-18. Although IL-18Rα^hi^ memory cells upregulated CD25 and produced IFN-γ, the IL-18Rα^lo^ exhausted cells failed to respond. Collectively, these findings indicate that as exhausted T cells adjust to the chronically infected environment, they lose their susceptibility to antigen-independent activation by cytokines, which compromises their ability to detect bacterial co-infections.

## Author Summary

Ongoing viral infections can corrupt the immune defense system. One way in which this occurs is by the induction of exhaustion in the host's anti-viral CD8 T cells, a key component of the immune response. Investigating the causes and consequences of T cell exhaustion will likely provide insights into potential strategies for improving immunity to ongoing infections. In addition, there is an increasing interest in determining how underlying chronic infections broadly impact immune responses to new infections. In this study we demonstrate that exhausted anti-viral T cells downregulate the receptor for the immune system factor IL-18. This downregulation is consequential as it prevents the function of exhausted cells from being rescued by certain combinations of immune system factors, cytokines, which potently activate conventional, highly protective responses. Moreover, the loss of this receptor disfavors the accumulation of anti-viral CD8 T cells during the initial phase of the response. We also show that whereas conventional anti-viral CD8 T cells can sense a bacterial co-infection that induces IL-12 and IL-18 production, the exhausted cells fail to respond. Collectively, these studies reveal how components of the immune defense system become recalibrated during an ongoing infection, altering their ability to respond to certain new infections.

## Introduction

Memory CD8 T cells typically develop following a short period of antigenic activation, which occurs during acute infections with intracellular pathogens. A hallmark of these memory T cells is their ability to rapidly respond following re-exposure to their original inducing antigen [reviewed in 1,2]. This recall response includes the production of cytokines such as interferon (IFN)-γ, the elaboration of cytotoxic effector activities, proliferation, and changes in the expression of cytokine receptors and adhesion molecules. In addition to their exquisite ability to respond to antigenic activation, memory CD8 T cells have also been shown to possess an innate-like ability to respond to certain sets of cytokines in the absence of antigen exposure [3]–[10]. Most notably, a combination of the proinflammatory cytokines IL-12 and IL-18 causes the pronounced production of IFN-γ by memory T cells [5], [6], [8]–[10]. Other cytokine combinations including IL-18 and IL-21, as well as IL-18 and type I IFN have been shown to have similar activating effects [3], [4], [11]. This sensitivity to cytokine stimulation endows memory T cells with the capacity to respond in an antigen- and T cell receptor (TCR)-independent manner to certain infections that induce inflammation, such as *Listeria monocytogenes* (LM) and *Burkholderia pseudomallei* [5], [6], [10]. Thus, pre-existing memory CD8 T cells can potentially contribute to the control of a broad set of infections due to their ability to detect changes in the inflammatory cytokine milieu.

During chronic viral infections the development of prototypic memory CD8 T cells is disrupted. Although initial CD8 T cell responses are usually elaborated, the responding virus-specific CD8 T cells undergo a differentiation process that results in their exhaustion [reviewed in 1,12,13]. The most severely exhausted CD8 T cells develop under conditions of high viral loads and ineffective CD4 T cell help. Although severely exhausted CD8 T cells retain expression of IFN-γ mRNA, they fail to produce IFN-γ protein after exposure to their cognate antigen [14], [15]. In addition, exhausted cells exhibit altered maintenance requirements, as they lose the self-renewal properties associated with normal memory T cells and may become deleted over time [16]–[18]. It is possible that exhaustion has evolved to allow antigen-specific T cells to become tuned to an environment of persisting antigen. Thus their loss of responsiveness to antigenic activation may serve as a safety mechanism that limits pronounced and sustained effector activities, which could be immunopathogenic. It is less clear, however, whether exhaustion alters the ability of T cells to mount antigen-independent responses to inflammatory cytokines.

Although T cell exhaustion has been described during several chronic viral infections including HIV and hepatitis C virus infections, it is most well characterized in mice persistently infected with lymphocytic choriomeningitis virus (LCMV). The LCMV system is particularly informative as different durations of infection can be established depending upon the isolate of virus and strains of mice used [17], [19]. In the current study we have used LCMV infection of mice to investigate whether the development of T cell exhaustion alters the ability of virus-specific CD8 T cells to perceive and respond to antigen-independent activation with combinations of IL-12, IL-18, and IL-21. The findings show that unlike effector and memory CD8 T cells, exhausted cells are not activated by these cytokines, and this correlates with differential expression of the IL-18-receptor-α (IL-18Rα). The decrease of IL-18Rα expression on exhausted CD8 T cells is consequential as it renders these cells more prone to deletion during the initial phase of persistent LCMV infection. In addition, lower IL-18Rα expression is associated with the failure of exhausted CD8 T cells to respond to bacterial co-infection by upregulating CD25 and producing IFN-γ.

## Results

### Effector and memory, but not exhausted, CD8 T cells respond to TCR-independent activation

For these studies we harnessed the LCMV system which provides an informative experimental platform for analyzing effector, memory, and exhausted CD8 T cell responses. We induced acute infections in C57BL/6 (B6) mice using the LCMV-Arm isolate which elicits a pronounced effector CD8 T cell response and subsequently establishes a long-lived pool of highly functional memory CD8 T cells following the complete resolution of the infection. By contrast, LCMV-cl13 infection of B6 mice results in a disseminated infection which is slowly brought under control over a period of several months, but continues to smolder in certain organs such as the kidney. This protracted infection induces an initial effector-like CD8 T cell response which is followed by the development of exhaustion. LCMV-cl13 infection of CD4^-/-^ mice is never brought under control and, although an initial effector-like response is mounted, severe CD8 T cell exhaustion develops as the chronic infection persists [17].

**Figure 1:**
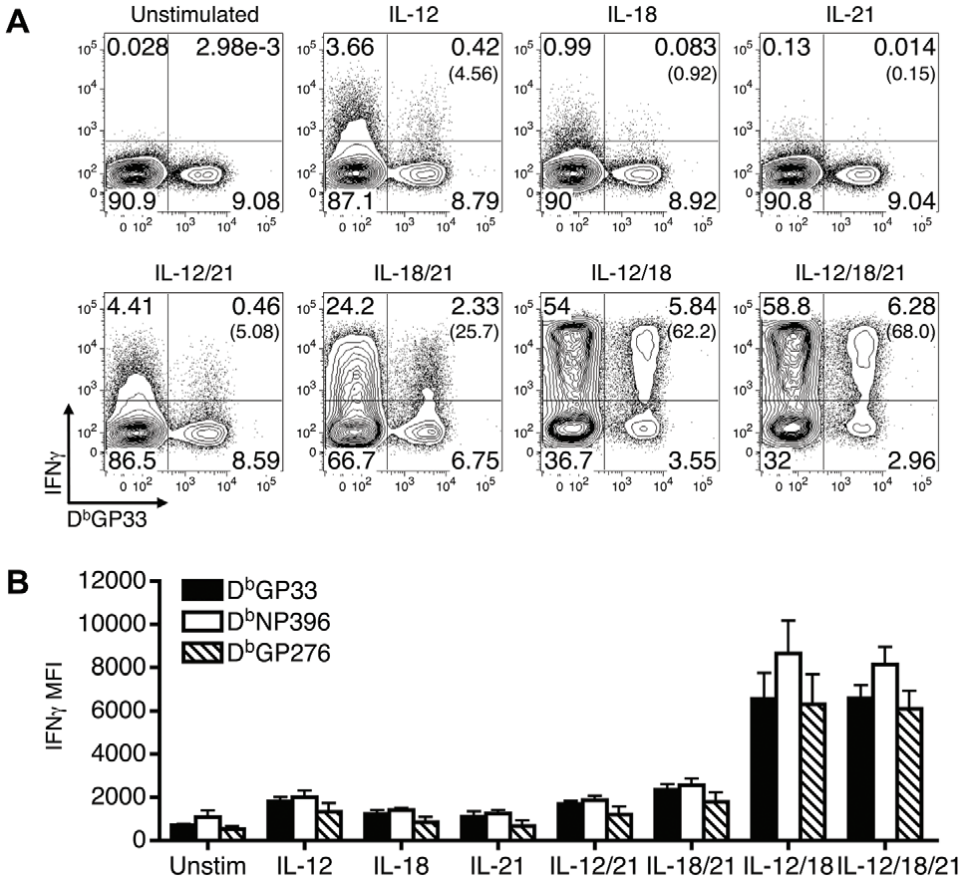
Qualitative and quantitative differences in IFN-γ production induced by combinations of IL-12, IL-18, and/or IL-21. Splenocytes from LCMV-Arm infected B6 mice (acute infection) were prepared at 9 days post-infection and stimulated with the indicated cytokines. (A) Representative flow cytometry plots show IFN-γ production by gated CD8 T cells following stimulation with the indicated cytokines. The numbers in parentheses indicate the percentage of IFN-γ-producing D^b^GP33^+^ cells. (B) The MFI of IFN-γ production by gated IFN-γ^+^ tetramer^+^ CD8 T cells is shown in response to stimulation with the various cytokines. Error bars are SD. Representative or composite results are shown from five experiments analyzing a total of 11-16 mice.

Previous studies have shown that various combinations of IL-12, IL-18, and IL-21 activate primed CD8 T cells to produce IFN-γ in the absence of antigenic stimulation [3]–[6], [8]–[11]. We confirmed and extended these findings using LCMV-specific effector CD8 T cells derived from B6 mice infected with LCMV-Arm (an acute infection) and observed qualitative and quantitative differences in the ability of the cytokine mixtures to stimulate IFN-γ production (Fig. 1). IL-12, IL-18, or IL-21 alone caused, at best, minimal IFN-γ production by LCMV GP33-specific effector CD8 T cells (Fig. 1A). IL-12+IL-18 with or without IL-21 stimulated IFN-γ production by 68±8% (SD) of the cells; however, IL-18+IL-21 had a more modest effect, activating 33±17% of these CD8 T cells, and the levels of IFN-γ production were also lower based upon the mean fluorescence intensity (MFI) of the IFN-γ^+^ population (Fig. 1B). IL-12+IL-21 was less effective at stimulating IFN-γ production (Fig. 1).

**Figure 2:**
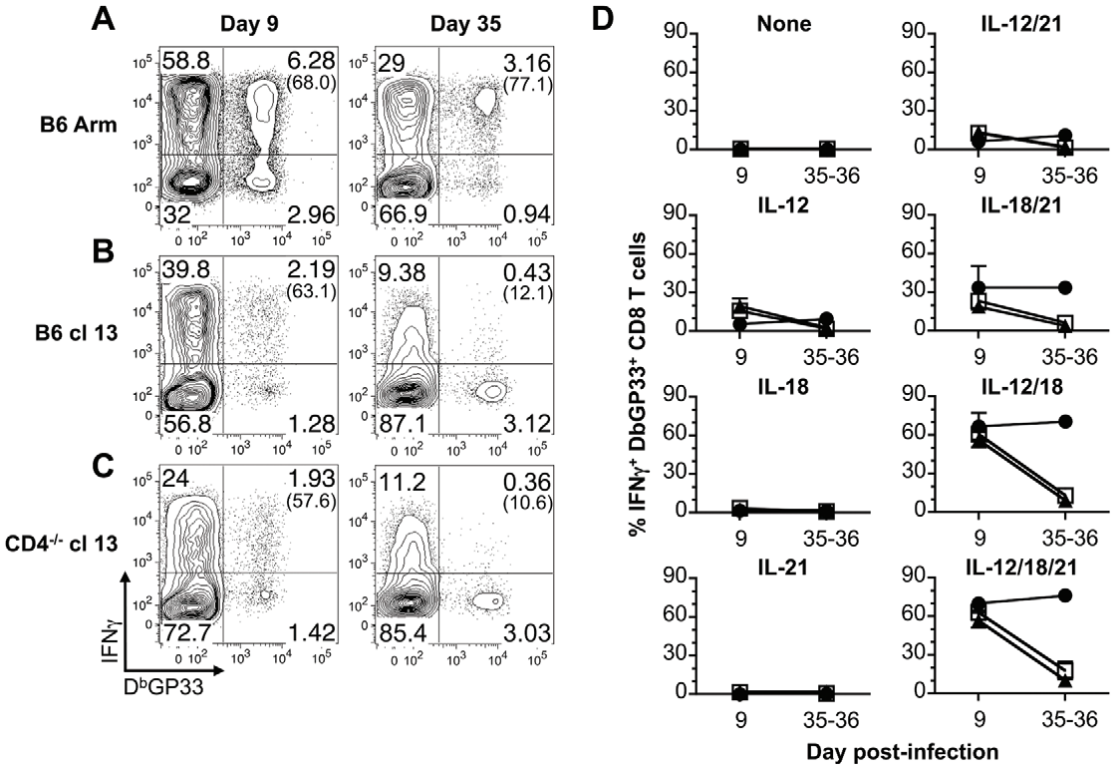
Exhausted CD8 T cells do not produce IFN-γ in response to antigen-independent stimulation. Splenocytes were stimulated for 5.5 hr with the indicated cytokines and IFN-γ production by D^b^GP33 tetramer binding cells was determined by intracellular cytokine analysis at days 9 (effector) or 35-36 (memory/exhausted) following infection. (A–C) Representative flow cytometry plots show gated CD8 T cells, and numbers in parentheses indicate the percentage of LCMV GP33-specific CD8 T cells that produce IFN-γ in response to activation with IL-12+IL-18+IL-21 following (A) acute, (B) protracted, or (C) chronic infection. (D) Mean percentage ± SD of LCMV GP33-specific CD8 T cells that produce IFN-γ in response to the indicated cytokines at days 9 and 35-26. Acute: LCMV-Arm, black circles; protracted: LCMV-cl13 infected B6 mice, white squares; and chronic: LCMV-cl13 infected CD4^-/-^ mice, black triangles. Representative (A) or composite (B) results are shown from five independent day 9 experiments with a total of 14-19 mice per group and three independent day 35-36 experiments with a total of 8-9 mice per group.

Since severely exhausted CD8 T cells fail to produce IFN-γ in response to stimulation with their cognate antigen [15]–[20], we next investigated whether effector, memory, and exhausted virus-specific CD8 T cells were susceptible to activation with the various cytokine combinations. To address this, acute (LCMV-Arm), protracted (LCMV-cl13 infection of B6 mice), and chronic (LCMV-cl13 infection of CD4^-/-^ mice) infections were established. During the effector phase of the CD8 T cell response, at day 9 following infection, LCMV GP33-specific CD8 T cells from all of the cohorts analyzed produced IFN-γ in response to a brief (5.5 hr) exposure to IL-12+IL-18 and IL-12+IL-18+IL-21 (Fig. 2). The other cytokine combinations and the single cytokines alone had minimal to modest stimulatory effects (Fig. 2D). By day 35 post-infection, the memory CD8 T cells that developed in acutely infected hosts retained the ability to respond to the cytokine combinations. By contrast, the development of exhaustion (days 35-36 post-infection of cl13 infected mice) was associated with the inability to produce IFN-γ in response to these cytokines (Fig. 2).

**Figure 3:**
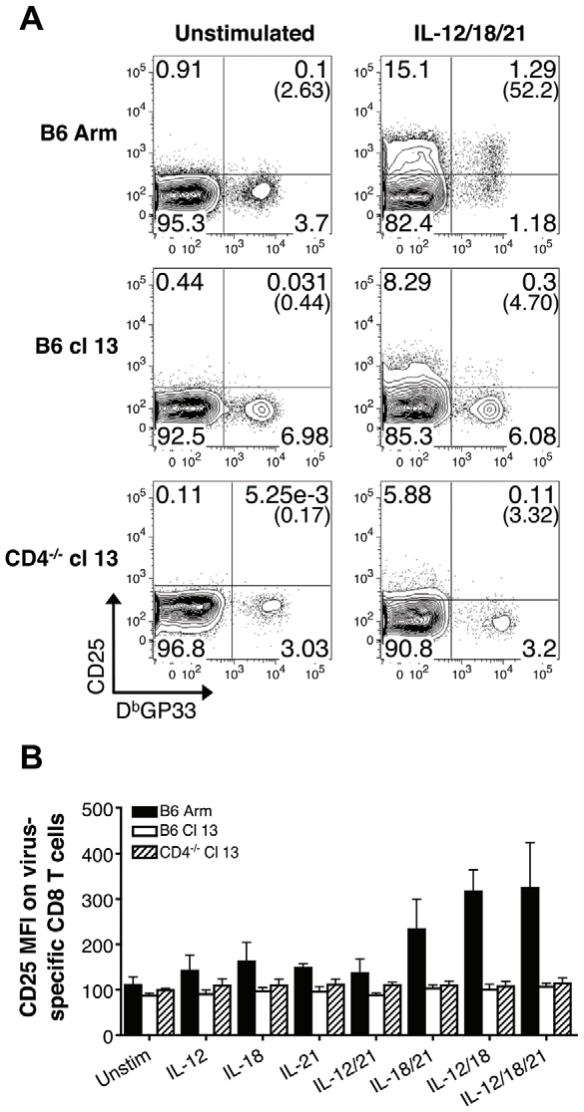
Exhausted CD8 T cells do not upregulate CD25 expression following IL-12, IL-18, and/or IL-21 exposure. (A) Representative flow cytometry plots show the expression of CD25 by gated CD8 T cells following acute (B6 Arm), protracted (B6 cl13), and chronic (CD4^-/-^ cl13) LCMV infections in response to a 5.5 hr incubation with or without IL-12, IL-18, and IL-21 in the absence of BFA. Numbers in parentheses indicate the percentage of CD25^+^ LCMV GP33-specific CD8 T cells. (B) The MFI± SD of CD25 expression on LCMV D^b^GP33-specific CD8 T cells following stimulation with the indicated sets of cytokines. Acute: LCMV-Arm, black bars; protracted: LCMV-cl13 infected B6 mice, white bars; and chronic: LCMV-cl13 infected CD4^-/-^ mice, striped bars. Representative or composite data are shown from two independent experiments at 38-39 days post-infection with a total of 7-8 mice per group.

We also checked whether exposure to IL-12, IL-18, and IL-21 either alone or in combination activated effector, memory, or exhausted CD8 T cells to upregulate expression of the IL-2 receptor-α chain, CD25. Changes in CD25 expression by LCMV-GP33 epitope-specific CD8 T cells were assessed following stimulation with the various cytokine combinations. The levels of CD25 increased on memory CD8 T cells from B6 LCMV-Arm infected mice following stimulation (Fig. 3). The magnitude of upregulation paralleled the trends observed for IFN-γ production with the most marked expression induced by IL-12+IL-18 and IL-12+IL-18+IL-21, while IL-18+IL-21 had less potent activating abilities (Fig. 2D and Fig. 3B). By contrast, LCMV-specific CD8 T cells from protracted and chronically infected mice failed to increase CD25 expression in response to stimulation with the cytokine panels (Fig. 3). Collectively these findings show the divergence between memory and exhausted CD8 T cells and demonstrate that persistent viral infections corrupt the ability of virus-specific CD8 T cells to respond to antigen-independent stimuli.

### Exhausted CD8 T cells downregulate IL-18Rα

**Figure 4:**
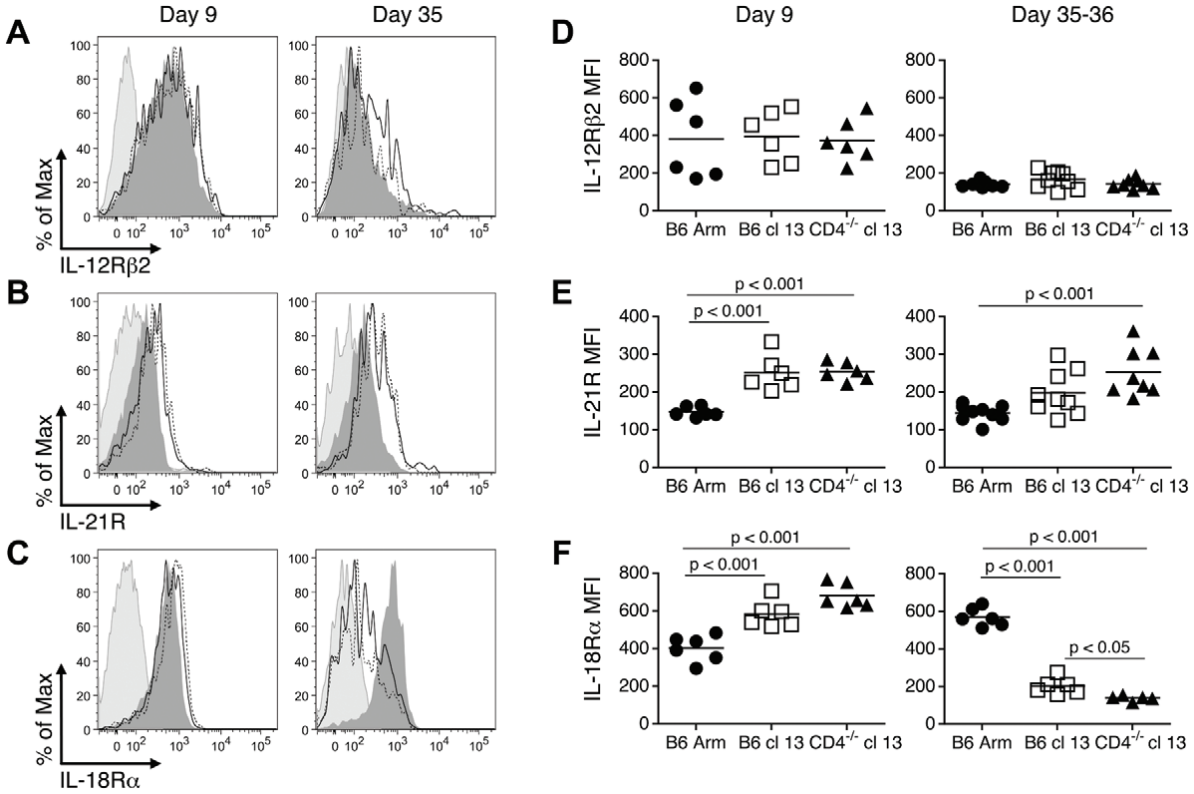
Cognate cytokine receptor expression on effector, memory, and exhausted CD8 T cells. Splenic LCMV D^b^GP33-specific CD8 T cells were analyzed at days 9 or 35-36 following acute, protracted, and chronic infections for the expression of IL-12Rβ2 (A, D), IL-21R (B, E), and IL-18Rα (C, F). (A–C) Representative histograms depict the expression of the cytokine receptors on GP33-specific CD8 T cells. Light shaded peak: isotype control, dark shaded peak: B6 Arm (acute), solid line: B6 cl13 (protracted), dotted line: CD4^-/-^ cl13 (chronic). (D-F) Show the MFI of cytokine receptor expression on the virus-specific CD8 T cells from individual mice. Mean values are indicated by the horizontal bars. The results are shown from a total of 6-9 individual mice per group, derived from 2-3 independent experiments.

To investigate why exhausted cells lose responsiveness to cytokine stimulation, the expression of the cognate cytokine receptors on virus-specific CD8 T cells was evaluated. Whereas the expression of IL-12Rβ2 was similar on LCMV GP33-specific CD8 T cells from mice undergoing acute, protracted, and chronic LCMV infections (Fig. 4A, D), the levels of the IL-21R tended to be higher in the protracted and chronically infected cohorts (Fig. 4B, E). The IL-18Rα was clearly expressed at high levels during the effector phase of the response (day 9) and was maintained on memory CD8 T cells (day 35, B6 Arm); however, the development of exhaustion in the protracted and chronically infected groups was associated with the downregulation of IL-18Rα (days 35-36, B6 cl13 and CD4^-/-^ cl13) (Fig. 4C, F).

**Figure 5:**
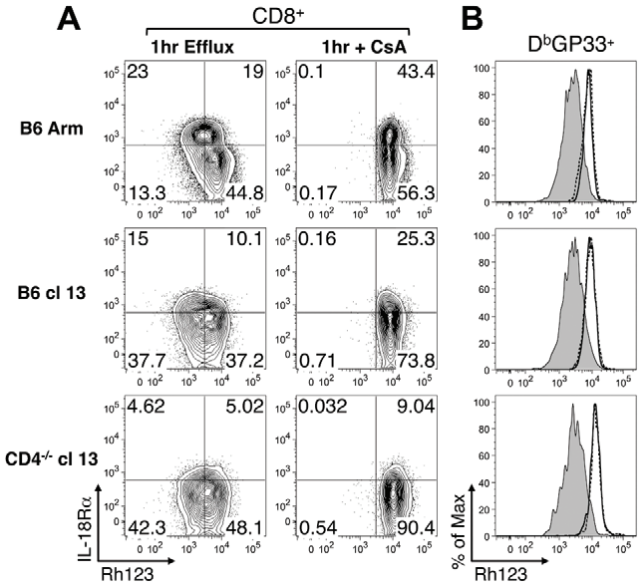
Rh123 efflux in memory and exhausted CD8 T cells. Splenocytes prepared from mice following acute (B6 Arm), protracted (B6 cl13), and chronic (CD4^-/-^ cl13) LCMV infections were loaded with Rh123 and then left untreated or treated with CsA or vinblastine. The efflux of Rh123 was determined 1hr later. (A) The Rh123 efflux profiles of gated CD8 T cells, without or with CsA treatment, are shown in conjunction with IL-18Rα staining. (B) The Rh123 efflux of gated LCMV GP33-specific CD8 T cells is shown. Shaded line: without inhibitor, solid line: with CsA, dashed line: with vinblastine. Data are representative of two independent experiments at day 38-39 post-infection with a total of 7-8 mice analyzed per group.

Efflux of the fluorescent dye rhodamine 123 (Rh123) is associated with populations of IL-18R^hi^ memory CD8 T cells which have self-renewing capabilities [21]. Since severely exhausted CD8 T cells are not always maintained over time [17]–[19] and express only low levels of the IL-18Rα, we next evaluated whether these cells were capable of effluxing Rh123. Consistent with previous findings [21], we found that IL-18Rα^hi^ CD8 T cells from acutely infected mice (Fig. 5A), which encompass the virus-specific memory T cell population (Fig. 5B), efficiently effluxed Rh123 during a one-hour period. This efflux of Rh123 was blocked by either Cyclosporine A (CsA; Fig. 5) or vinblastine (Fig. 5B), which inhibit ABCB1 transporters required for the removal of Rh123 [21], [22]. By contrast, virus-specific CD8 T cells from protracted (B6 cl13) and chronically (CD4^-/-^ cl13) infected mice, which have downregulated the IL-18Rα, effluxed Rh123 at least as efficiently as their memory counterparts from acutely infected mice (Fig. 5). Thus, even though exhausted CD8 T cells have altered maintenance requirements and proliferative properties, in chronic LCMV infection the levels of IL-18Rα on virus-specific CD8 T cells do not correlate with their ability to efflux Rh123.

### IL-18Rα expression influences the maintenance of CD8 T cells during the contraction phase

To further examine the significance of decreased IL-18Rα expression on virus-specific CD8 T cell responses, we analyzed bone marrow chimeras generated by reconstituting lethally irradiated mice with a mixture of CD45.1 IL-18Rα^+/+^ cells and either CD45.2 IL-18Rα^-/-^ (experimental) or CD45.2 IL-18Rα^+/+^ (control) cells. By 8 weeks following reconstitution, the mean proportion of CD8 T cells that were IL-18Rα^-/-^ (CD45.2) was 59% with a range of 53-64% in the experimental chimeras, and in the control chimeras the fraction of CD8 T cells that were CD45.2 (IL-18Rα^+/+^) was 42% with a range of 35-46%.

**Figure 6:**
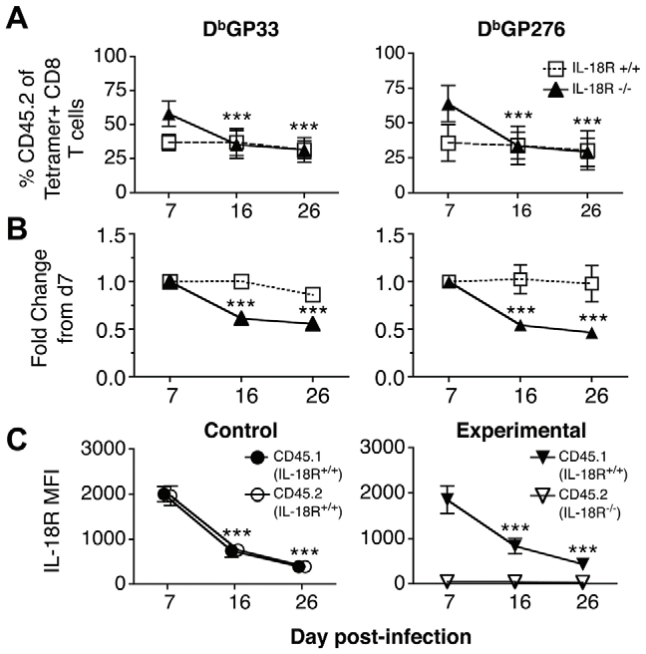
LCMV-specific IL-18Rα^-/-^ CD8 T cells are preferentially lost from day 7-16 following cl13 infection. The maintenance of IL-18Rα^+/+^ and IL-18Rα^-/-^ LCMV GP33-specific CD8 T cells was monitored in mixed bone marrow chimeras following cl13 infection. Mice from control (CD45.1, IL-18Rα^+/+^ & CD45.2, IL-18Rα^+/+^) and experimental (CD45.1, IL-18Rα^+/+^ & CD45.2, IL-18Rα^-/-^) cohorts were bled and analyzed at the indicated days post-infection. (A) The percentage of CD45.2 LCMV GP33-specific CD8 T cells over time and (B) the fold change in the CD45.2^+^GP33^+^ population normalized to the levels detected at day 7 post infection in the control (IL-18Rα^+/+^, open squares) and experimental (IL-18Rα^-/-^, filled triangles) cohorts. (C) The MFI of IL-18Rα expression on CD45.1^+^ and CD45.2^+^ GP33-specific cells in the control and experimental cohorts. Data are shown from two independent experiments with (A and B) 8-11 mice per group and (C) 3-10 mice per group at each time-point; *** p<0.001.

Both the experimental and control chimeras responded to infection with LCMV-cl13. By 7 days following infection the mean proportion of CD8 T cells that were CD45.2 (IL-18Rα^-/-^) was 53% with a range of 38-63% in the experimental chimeras, and in the control chimeras the fraction of CD8 T cells that were CD45.2 (IL-18Rα^+/+^) was 39% with a range of 31-46%. Virus-specific CD45.2^+^ CD8 T cell responses also became detectable in both cohorts (Fig. 6A) and the fraction of CD45.2 tetramer binding cells at this initial time point essentially reflect the pre-infection degree of chimerism. Between days 7-16 following infection, the virus-specific IL-18Rα^-/-^ (CD45.2) cells appeared to preferentially contract in the experimental chimeras, as the fraction of these cells decreased during this period. However, the proportion of virus-specific CD8 T cells that were CD45.2 (IL-18Rα^+/+^) in the control chimeras remained stable (Fig. 6A). Although the IL-18Rα^-/-^ CD8 T cells appeared to be outcompeted during the second week of infection, this disproportionate loss stabilized between days 16-26 (Fig. 6A, B). This stabilization was concurrent with the downregulation of IL-18Rα on the D^b^GP33^+^CD8 T cells in the control and experimental chimeras (Fig. 6C).

### Memory but not exhausted CD8 T cells respond to a bystander bacterial infection

**Figure 7:**
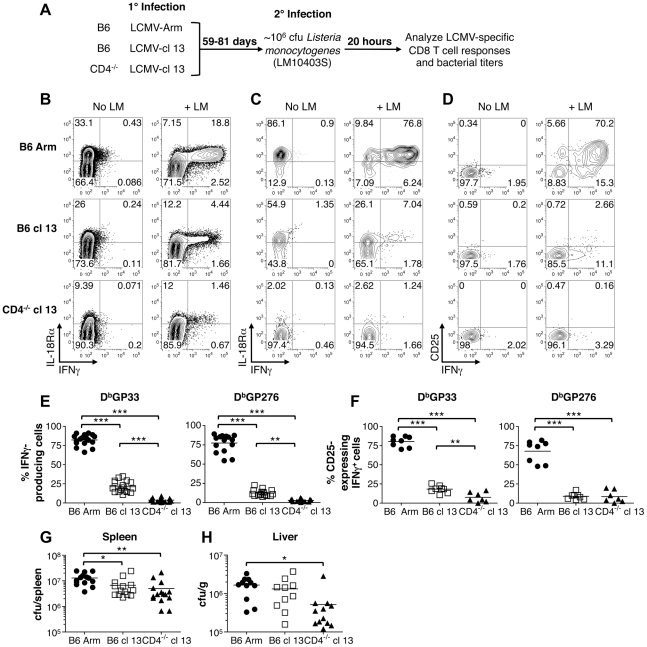
Memory but not exhausted T cells express IFN-γ and CD25 in response to LM infection. Cohorts of mice were challenged with wild-type LM 59-81 days following acute (LCMV-Arm), protracted (LCMV-cl13 infected B6 mice) and chronic (LCMV-cl13 infected CD4^-/-^ mice) infections. Splenocytes were prepared 20 hr after the bacterial infection and cultured in the presence of BFA for 3hr prior to surface MHC tetramer staining and intracellular staining for IFN-γ accumulation. (A) Overview of the experimental design. (B) Representative flow cytometry plots show IL-18Rα expression and IFN-γ production by gated CD8 T cells are shown with and without LM infection. (C) IL-18Rα and (D) CD25 expression in conjunction with IFN-γ production are shown for gated LCMV D^b^GP33^+^ CD8 T cells. The percentages of (E) IFN-γ-producing and (F) CD25-expressing IFN-γ^+^ LCMV D^b^(GP33) and D^b^(GP276) epitope-specific CD8 T cells are shown for individual mice, and mean values are indicated by the horizontal bars. (G) and (H) Bacterial titers in the spleens and livers, respectively, following LM challenge of the cohorts of mice that were previously infected with LCMV. Flow cytometry plots show representative data from either (B, C) three or (D) two independent experiments analyzing a total of 11-12 LM challenged mice per group. Graphs show results from (E) 4–5 experiments with 15-18 mice per group, from (F) two experiments with 6-8 mice per group, from (G) four experiments with 14-15 mice per group, and from (H) three experiments with 10–12 mice per group. *** p<0.001, ** p<0.01, * p<0.05.

Given the marked downregulation of IL-18Rα on exhausted CD8 T cells, we evaluated whether this would compromise their ability to respond in an antigen-independent manner to a bacterial co-infection. LM has been previously shown to stimulate IFN-γ production by memory CD8 T cells that are not specific for LM antigens but respond due to their sensitivity to IL-12 and IL-18 induced by the bacterial infection [6]. Thus to provide a stringent in vivo readout of whether the innate-like responses of exhausted CD8 T cells were corrupted, cohorts of acute, protracted, or chronically infected mice were challenged with wild-type LM, which does not encode any known LCMV epitopes. We used a challenge inoculum of 10^6^ cfu as this dose has been previously shown to induce a pronounced IFN-γ^ +^ response by memory CD8 T cells [6], and evaluated the impact of this co-infection on the “bystander” LCMV-specific CD8 T cells 20hr later (Fig. 7A). In all cases, the CD8 T cells that became IFN-γ^+^ following LM challenge were IL-18Rα^hi^, indicating that the ability to sense this proinflammatory cytokine was critical for the response (Fig. 7B, C). In the acutely infected group LM infection caused between 66-91%, of LCMV GP33-specific memory CD8 T cells to produce IFN-γ (Fig. 7E). This response was severely curtailed in hosts undergoing protracted or chronic LCMV infections as only 11-35% and 0.9-9%, respectively, of the GP33-specific CD8 T cells became IFN-γ positive following LM co-infection, and similar trends were observed for the GP276 viral epitope-specific responses (Fig. 7E). We further checked whether LM infection also resulted in increased CD25 expression on the LCMV-specific CD8 T cells. Again, although the memory CD8 T cells did upregulate CD25, the responsiveness of the exhausted cells was significantly diminished, which was consistent with the observations of IFN-γ production (Fig. 7D, F). Thus, whereas memory CD8 T cells can vigorously respond to sets of inflammatory cytokines and certain bacterial infections, the development of exhaustion is associated with downregulation of IL-18Rα and the inability to respond to both the underlying viral infection and the inflammatory cytokine milieu. Despite this clear cut defect in the innate-like properties of virus-specific CD8 T cells in the protracted and chronically infected cohorts, by 20 hr following LM infection bacterial titers were somewhat lower in the spleens (Fig. 7G) and livers (Fig. 7H) of these mice. This paradoxical finding indicates that although exhausted CD8 T cells lose their ability to respond to changes in the levels of inflammatory cytokines induced by the bacterial challenge, other immunological alterations in the chronically infected host help confer resistance to secondary infections.

## Discussion

Our investigation of the sensitivity of virus-specific CD8 T cells to stimulation with combinations of IL-12, IL-18, and IL-21 highlights the divergence between memory and exhausted T cells [1], [12], [13]. The effector populations that develop during the early stages of acute, protracted, and chronic LCMV infection are more similar, and in all cases a substantial fraction express the IL-18Rα and produce IFN-γ as well as increase expression of CD25, in response to antigen-independent stimulation by the cytokines tested. Distinct differences manifest as constituents of the effector pool transition into the memory compartment following resolution of the acute infection, and as the exhausted state progressively develops if the infection persists. The memory cells are clearly distinguished by the maintenance of IL-18Rα expression and retain the ability to respond to activation by specific cytokines in the absence of antigen. By contrast, as exhausted CD8 T cells emerge in persistently infected mice, the IL-18Rα is downregulated. This mirrors the reduction in IL-18Rα observed on HIV-specific CD8 T cells, suggesting that decreased levels of IL-18Rα is a common feature of exhaustion [23].

As shown in this report, the loss of IL-18Rα on exhausted CD8 T cells correlates with their failure to prominently react to both in vitro activation with specific cytokine combinations and also to in vivo exposure to bystander LM infection. Although bona fide memory CD8 T cells are highly sensitive to both TCR triggers and cytokine mediated signals that induce IFN-γ, the well-described inability of severely exhausted T cells to produce marked amounts of IFN-γ in response to TCR-dependent antigenic activation [12], [13], [15], [17]–[19] is not rescued by cytokine exposure. Virus-specific memory CD8 T cells not only respond to cytokine activation and bystander LM infection by producing IFN-γ but also upregulate expression of the IL-2Rα chain, CD25. The synergistic ability of IL-18 in combination with either IL-12 or IL-21 to induce CD25 may serve to pre-trigger memory CD8 T cells encountering an inflammatory environment and other helper mediators, and this possibly augments their proliferation and reacquisition of effector traits if antigenic signals are also received. This response is abolished in exhausted CD8 T cells and likely further impedes their responsiveness to either ongoing antigenic activation or co-infection associated danger signals. Thus, as virus-specific T cells succumb to exhaustion, they adjust to the sustained presence of antigen as well as the unique cytokine milieu that occurs in the persistently infected host, and appear to undergo a global desensitization in their ability to respond to effector function-inducing signals.

The upregulation of IL-18Rα transcript levels occurs during the contraction phase of the response, and several studies have shown that memory T cells express high levels of IL-18Rα [14], [23], [24]. Nevertheless, memory T cell formation and maintenance does not require IL-18 signals as the kinetics, magnitudes, and longevity of LM-specific CD8 T cell responses in IL-18- and IL-18Rα- deficient mice resemble those observed in immunocompetent animals [25]. During chronic LCMV infection, exhausted T cells form and also remain present even though expression of IL-18Rα becomes downregulated. Our analyses of mixed bone marrow chimeras show that although both IL-18Rα^+/+^ and IL-18Rα^-/-^ CD8 T cells do initially participate in the response to LCMV cl13 infection, the virus-specific IL-18Rα^-/-^ CD8 T cells are outcompeted by their IL-18Rα^+/+^ counterparts following the peak of the effector phase of the response. The proportion of IL-18Rα^-/-^ T cells subsequently stabilizes, and this coincides with the decrease in receptor expression on the index IL-18Rα^+/+^ population. The apparent preferred maintenance of IL-18Rα^+/+^ CD8 T cells during the contraction phase of the response is consistent with the documented pro-survival functions of IL-18 and its roles in limiting activation-induced cell death [26]. IL-18 has also been shown to increase the proliferation of memory CD8 T cells as they mount a recall response upon reencountering presented antigen [27]. In chronically infected mice viral antigen is present throughout the response, and therefore it is plausible that in this setting IL-18 exerts a dual survival and proliferative role. These effects are temporally limited due to the progressive downregulation of IL-18Rα that occurs as exhaustion develops.

The prototypic memory CD8 T cells and exhausted T cells that are present following the contraction phase of the response have distinct maintenance requirements [28], [29]. Memory CD8 T cells are maintained at remarkably stable levels in the absence of antigen, principally due to the common-γ chain cytokine family members IL-7 and IL-15; however, the cognate receptors for these homeostatic cytokines are downregulated on exhausted CD8 T cells [28]–[31]. Instead, the continued presence of viral antigen appears to preserve the exhausted CD8 T cells and allows them to bypass the normal necessity for IL-7 and IL-15. Interestingly, in humans expression of the IL-18Rα is linked with the ability of memory phenotype cells to reconstitute and “self-renew” following chemotherapy, and this property is also associated with the capacity of these cells to efflux Rh123 [21]. We confirmed this finding in acutely infected mice as the ability to efflux Rh123 was primarily detected in the IL-18Rα^hi^ subset of cells; however, the IL-18Rα^lo^ exhausted T cells were also similarly capable of effluxing Rh123. This differs from the observations of Turtle *et al.* [21], but likely reflects the unique properties of exhausted T cells, which are maintained despite downregulation of the IL-18Rα and retain the ability to efflux Rh123.

The establishment of virus-specific memory CD8 T cells which can detect and respond to increased levels of inflammatory and regulatory cytokines allows them to provide a broader level of immunological protection that is not governed by their precise antigen-specificity [5]–[7], [10]. This is well described following LM infection of mice as memory CD8 T cells specific for non-LM encoded epitopes confer some level of protection against bacterial challenge [6], [10]. We document clear differences in the ability of acute and chronic infections to elicit virus-specific CD8 T cell populations capable of detecting bystander LM infections. Our findings show that LCMV-specific memory CD8 T cells from acutely infected mice mount a marked bystander response to LM infection resulting in the production of IFN-γ and upregulation of CD25. By contrast, the ability of exhausted virus-specific CD8 T cells to mount a non-antigen specific, innate-like, response to a bacterial co-infection is severely curtailed. The most pronounced exhaustion of CD8 T cells develops during chronic infection in the absence of sufficient CD4 T cell help. During HIV infection there is a profound loss of CD4 T cells in the gastrointestinal tract [32]–[34]. This is associated with translocation of microbial flora and products from the intestinal lumen and the detection of lipopolysaccharides and bacterial DNA in the plasma, which results in chronic immune activation and accelerated disease progression [35], [36]. Therefore, it will be interesting to dissect inter-relationships between viral loads, CD8 T cell exhaustion, and the extent of microbial translocation following HIV infection, as well as during pathogenic and non-pathogenic SIV infections.

There is increasing interest in understanding how underlying persistent viral infections have a generalized impact on immune responses [37]. Commonly, individuals are exposed to a myriad of infections and the collective induction or suppression of immune system functions caused by the “virome” likely mold the formation of subsequent responses. Although the innate-like response of exhausted LCMV-specific CD8 T cells to LM co-infection was significantly reduced, the bacterial burden in the LCMV chronically infected cohort was either no different or lower than that detected in the acutely infected group. This paradoxical finding suggests that other immunological attributes of the chronically infected host contribute to overall resistance or susceptibility to secondary or opportunistic infections. For example, macrophage activation caused as a result of acute LCMV infection has been proposed to account for increased resistance to LM infection [38]. Similarly macrophage activation in mice persistently infected with either murine γ-herpesvirus 68 or mouse cytomegalovirus confers protection against LM and *Yersinia pestis* challenge [39]. Conversely, other immunological alterations, such as ablation of type I IFN production by plasmacytoid dendritic cells, which occurs during chronic LCMV infection, can promote establishment of certain opportunistic infections [40]. The development of exhaustion associated with chronic LCMV infection clearly results in a profound disruption of usually highly responsive and effective anti-viral T cells. These alterations impact both their ability to respond to antigenic stimuli as well as inflammatory cytokines. Such changes in the properties of T cells occur in the context of global shifts in immune system functions which emerge as the hosts adjusts to the ensuing chronic infection, including disturbances in splenic architecture, and alterations in the composition and activation status of classic innate immune effectors. The collective changes in the immunological environment that occur likely act in concert to limit bacterial growth following LM challenge, compensating for the dysregulation in the innate-like properties of the virus-specific CD8 T cell population.

Overall, the signature loss of IL-18Rα expression by exhausted virus-specific CD8 T cells represents one mechanism of re-calibrating the cellular immune response to ongoing chronic infection. This phenotypic shift likely represents one of many evolutionary adaptations that help prevent severe immunopathology. Nevertheless, the combined alterations to the host response caused by virus-persistence may help or hinder resistance to new or current infections.

## Materials and Methods

### Ethics statement

All procedures with experimental mice were approved by the University of Alabama at Birmingham Institutional Animal Care and Use Committee in accordance with NIH guidelines.

### Mice and infections

C57BL/6J (B6), C57BL/6-Cd4^tm1Mak^/J (CD4^-/-^), B6.SJL-Ptprc^a^Pepc^b^/BoyJ (CD45.1), B6.129S7-Rag1^tm1Mom^/J (Rag-1^-/-^), and B6.129P2-Il18r1^tm1Aki^/J (IL-18Rα^-/-^) mice were originally purchased from Jackson Laboratory (Bar Harbor, ME). All mice were bred and/or maintained in fully accredited facilities at the University of Alabama at Birmingham. For acute infections mice were infected by i.p. injection with 2×10^5^ PFU LCMV-Armstrong (Arm). Protracted and chronic infections were established by i.v. inoculation with 2–4×10^6^ PFU LCMV-clone 13 (cl13) into B6 and CD4^-/-^ mice, respectively [17]. In certain experiments 0.96–3.3×10^6^ colony-forming units (CFU) of the 10403S strain of *Listeria monocytogenes* (LM) (kindly provided by Dr. D. Portnoy, University of California, Berkeley, CA) was administered by i.v. injection into mice that had been infected with LCMV 59-81 days previously. To determine LM titers 20 hr following co-infection, suspensions of splenocytes were diluted with an equal volume of 0.5% Triton X-100 (Fisher Scientific, Fair Lawn, NJ). Livers were collected into PBS, weighed, diluted with an equal volume of 0.5% Triton X-100 and then homogenized [essentially as in 41]. Numbers of CFU were determined by plating serial dilutions on BHI agar plates.

### Cell preparation

Splenocytes and blood samples were processed as previously described [42]. For LM co-infection studies splenic samples were collected, prepared, and cultured for 3hr at 37°C in medium without antibiotics but containing 10 µg/ml Brefeldin A (Sigma-Aldrich, St. Louis, MO) prior to staining and flow cytometric analyses [6].

### In vitro stimulations

Splenocytes were cultured for 5.5 hr at 37°C in the presence or absence of recombinant murine IL-12 (Biosource/Invitrogen, Camarillo, CA or Peprotech, Rocky Hill, NJ), IL-18 (Biosource/Invitrogen, Camarillo, CA), IL-21 (R&D Systems, Minneapolis, MN or Peprotech, Rocky Hill, NJ), or various combinations of the three cytokines. All cytokines were used at a final concentration of 20 ng/ml. Brefeldin A (Golgi Plug, BD Biosciences, San Jose, CA) was added for the last 1.5 hr of culture to facilitate the intracellular accumulation of IFN-γ.

### Cell staining and flow cytometry

Surface and intracellular staining was performed essentially as previously described [17]. All samples were pre-treated with anti-CD16/CD32 mAb (clone 2.4G2) (UAB Immunoreagent Core) prior to staining. Surface staining was performed using various combinations of anti-CD44 (clone IM7, BD Biosciences), anti-CD25 (clone 3C7 or PC61, Biolegend, San Diego, CA), anti-IL-12Rβ2 (clone 305719, R&D Systems), anti-IL-18Rα (clone 112614, R&D Systems), and anti-IL-21R (clone 4A9, Biolegend) mAbs together with anti-CD8α clone 53-6.7 (eBioscience), and PE or allophycocyanin conjugated MHC class I tetramers. MHC tetramers were produced in house or obtained from the National Institute of Allergy and Infectious Diseases tetramer core facility, Atlanta, GA. For intracellular staining the anti-IFN-γ antibody XMG1.2 was used (eBioscience). All samples were acquired on an LSRII flow cytometer (BD Biosciences), and data were analyzed using FlowJo software (Tree Star, Ashland, OR).

### Rhodamine 123 efflux

Splenocytes were loaded with Rhodamine 123 (Rh123) (Invitrogen or Sigma-Aldrich) in RPMI-1640 supplemented with 10% FCS, 50 µM β-mercaptoethanol, 100 U/ml penicillin, and 100 µg/ml streptomycin for 30 minutes on ice. Samples were then washed and cultured for 1hr at 37°C in the absence or presence of either 0.1 µg/ml Cyclosporine A (CsA) (Sigma-Aldrich) or 5 µg/ml Vinblastine (Sigma-Aldrich). After incubation cells were washed and stained for CD8α and IL-18Rα together with PE-conjugated D^b^GP33 MHC tetramers, as described above. Samples were resuspended in 0.1% BSA and 0.01% NaN_3_ in PBS prior to flow cytometric analyses, and Rh123 fluorescence was detected using a 530/30 bandpass filter.

### Mixed bone marrow chimeras

Chimeras were generated essentially as previously described [42]. Bone marrow from CD45.1 (IL-18Rα^+/+^), CD45.2 (IL-18Rα^-/-^), and CD45.2 (IL-18Rα^+/+^) B6 mice was T cell depleted using anti-CD5 (Ly-1) microbeads (Miltenyi Biotec, Auburn, CA). Recipient Rag-1^-/-^ mice were administered a split dose of radiation to give a total exposure of ∼1000 rads. These recipient mice were then reconstituted by i.v. injection with an approximate 50∶50 ratio (1.4×10^6^ total cells) of either CD45.1 (IL-18Rα^+/+^): CD45.2 (IL-18Rα^+/+^) bone marrow, to generate a control cohort, or CD45.1 (IL-18Rα^+/+^): CD45.2 (IL-18Rα^-/-^) bone marrow, to generate an experimental cohort. The mice were infected with 4×10^6^ PFU cl13 at 9 or 11 weeks after reconstitution.

### Statistical analysis

One-way ANOVA was used to determine statistical significance. P values were calculated using Prism software (Graph Pad, San Diego, CA). Statistical significance is defined as *p<0.05, **p<0.01, ***p<0.001.
